# Predicting protein-protein binding sites in membrane proteins

**DOI:** 10.1186/1471-2105-10-312

**Published:** 2009-09-24

**Authors:** Andrew J Bordner

**Affiliations:** 1 Mayo Clinic, 13400 East Shea Boulevard, Scottsdale, AZ 85259, USA

## Abstract

**Background:**

Many integral membrane proteins, like their non-membrane counterparts, form either transient or permanent multi-subunit complexes in order to carry out their biochemical function. Computational methods that provide structural details of these interactions are needed since, despite their importance, relatively few structures of membrane protein complexes are available.

**Results:**

We present a method for predicting which residues are in protein-protein binding sites within the transmembrane regions of membrane proteins. The method uses a Random Forest classifier trained on residue type distributions and evolutionary conservation for individual surface residues, followed by spatial averaging of the residue scores. The prediction accuracy achieved for membrane proteins is comparable to that for non-membrane proteins. Also, like previous results for non-membrane proteins, the accuracy is significantly higher for residues distant from the binding site boundary. Furthermore, a predictor trained on non-membrane proteins was found to yield poor accuracy on membrane proteins, as expected from the different distribution of surface residue types between the two classes of proteins. Thus, although the same procedure can be used to predict binding sites in membrane and non-membrane proteins, separate predictors trained on each class of proteins are required. Finally, the contribution of each residue property to the overall prediction accuracy is analyzed and prediction examples are discussed.

**Conclusion:**

Given a membrane protein structure and a multiple alignment of related sequences, the presented method gives a prioritized list of which surface residues participate in intramembrane protein-protein interactions. The method has potential applications in guiding the experimental verification of membrane protein interactions, structure-based drug discovery, and also in constraining the search space for computational methods, such as protein docking or threading, that predict membrane protein complex structures.

## Background

Integral membrane proteins constitute a significant fraction of all proteins in sequenced organisms and also are targets of slightly more than half of all current drugs [1,2]. Similar to non-membrane proteins, many membrane proteins form complexes in order to carry out their biological function. Structural details of these protein-protein interactions can aid in generating experimentally verifiable mechanistic hypotheses for the relevant complexes and also can form a basis for the structure-based discovery of therapeutics to modulate these interactions. However, high-resolution experimental structures of membrane protein complexes are relatively scarce (< 1% of all Protein Data Bank structures), due to technical difficulties in obtaining X-ray or NMR structures [3]. Also, even with an available structure, the annotation of the biological complex in the Protein Data Bank (PDB) file may be incorrect [4]. Furthermore, even as new techniques are developed to speed up the experimental determination of membrane protein structures, the combinatorial nature of protein-protein interactions precludes solving the structures of all possible protein complexes from an organism's proteome.

Computational methods can address these challenges by providing predictions of which residues on the protein surface participate in protein-protein interactions. These predictions can be subsequently verified by, for example, mutagenesis experiments. The predictions can also be used as constraints for predicting the structure of the protein complex by, for example, protein-protein docking. Existing computational methods for predicting protein-protein binding sites can be broadly classified into those that utilize only 1D sequence information and those that require some information about the 3D protein structure. Sequence-only methods [5-8] have the advantage that they can be applied to proteins for which no experimental structures are available and no close templates can be found for comparative modeling. However, structure provides additional information that helps distinguish binding site residues, such as solvent accessibility and the proximity of residues in 3D space. Because of these additional signals, prediction methods that incorporate this information generally perform better than sequence-only methods, although the use of different data sets and interface residue definitions prevents a direct comparison. Many previous structure-based methods used either scoring functions [9-11], artificial neural networks (ANNs) [12-15], or Support Vector Machines (SVMs) [16-18] trained on various properties within roughly circular surface patches to predict protein-protein binding sites. Two exceptions are a study that limited the predictions to surface pockets [19] and a recent study that used a Random Forest trained on residue types and properties within a sliding 9-residue window for prediction [20].

Here we consider the problem of predicting protein-protein binding sites within the intramembrane region of integral membrane proteins. The previous studies mentioned above were limited to non-membrane proteins, for which considerably more experimental structures are available. Nonetheless, we find that there are currently a sufficient number of structures for training and validating a predictor that achieves accuracy comparable to our previous results for non-membrane proteins [18]. There are large differences in the frequencies of residue types on the surfaces of membrane and non-membrane proteins due to their hydrophobic and hydrophilic environments, respectively. This means that separate predictors, trained only on data from their respective class of proteins (membrane or non-membrane), are needed. The prediction method employs a Random Forest trained on residue frequencies in a multiple alignment of related protein sequences and the evolutionary rates of each site. Random Forest predictions are first made for individual surface residues and then these are averaged over a local surface region in order to arrive at the final prediction. This procedure was found to yield better accuracy than directly including the properties of surrounding residues in the training data, as was done in previous machine learning based methods. In addition, we compared the residue properties between protein-protein binding sites and the remaining surface and also between membrane and non-membrane proteins in order to discern which properties contribute to the prediction in each case. Also, we examined the relative contribution of each property to the overall prediction accuracy and considered examples of predictions for particular membrane proteins.

## Methods

### Benchmark set of membrane protein complex structures

A diverse set of alpha-helical membrane protein complex structures was first compiled for training and testing the prediction method. Monomers as well as multimeric complexes were included. The initial set of PDB entries for alpha-helical membrane proteins were taken from the PDBTM database [21,22]. A non-redundant subset of protein complexes, for which no pair of complexes have all proteins differing by less than 30% sequence identity, were then selected from each initial set of structures. Information on generating the biological complex in the PDB structure files (the BIOMT record) was used as an initial guess of the complex structure. Because this information is sometimes erroneous [4,23], it was compared with the literature and the structure of the complete protein complex was corrected where necessary.

Next, a set of non-redundant proteins, each of which contacts at least one other protein in a complex, was extracted from these structures. Because the individual proteins are taken from structures of protein complexes, their protein-protein binding sites are known. This set of protein structures was then used to train the prediction method and to assess its accuracy. The same procedure was also used to build a set of beta barrel membrane protein complex structures as well as a non-redundant set of proteins taken from these complexes with known protein-protein binding sites. Finally, the alpha-helical and beta barrel sets were combined to make the membrane protein benchmark set.

The final set of membrane protein complexes contained 64 alpha-helical multimeric protein complexes comprised of 149 unique subunits, 17 alpha-helical monomeric complexes, 14 beta barrel homomultimeric complexes, and 23 beta barrel monomers. The details of this benchmark set are provided as additional file 1  accompanying this article.

### Training data

Only surface residues, with relative solvent accessible surface area (SASA) ≥ 0.2, that are also within the hydrophobic core of the membrane are considered and so included in the training data. The relative SASA is calculated by dividing the residue SASA by the value for the same residue type in an extended conformation surrounded by glycine residues. Residues in the membrane core have z-coordinates with |*z*| ≤ 15 Å, in which the z-axis is perpendicular to the plane of the membrane predicted by PDBTM and the origin is in the center of the membrane. In other words, the membrane core was assumed to be 30 Å thick, which is in agreement with the approximate values from PDBTM predictions and experimental results on lipid bilayers [24].

Random Forest predictions were made for each individual residue based on its properties. The training data for each residue consisted of frequencies of each of the 20 standard residues in a multiple sequence alignment of similar sequences and the evolutionary rate. The sequence alignments were created by searching for similar protein sequences in the NCBI nr database with BLAST [25] at an E-value cutoff of 10^-2^, removing redundant sequences at the 90% sequence identity level using the CD-HIT program [26], and generating multiple alignments of the remaining sequences with MUSCLE [27]. Only proteins with at least 20 sequences in the final alignment were included in the training set. This criterion reduced the number of unique proteins included in the training data to 128. The residue frequency for a particular residue type was simply calculated as the fraction of residues of that type in the corresponding multiple sequence alignment column. The evolutionary rate, which varies inversely with conservation, was calculated using the REVCOM method [28]. Because REVCOM accounts for the evolutionary relationships between the protein sequences via an inferred phylogenetic tree, the resulting evolutionary conservation values are more robust to the particular set of sequences and local alignment errors than methods that do not, such as the column entropy. Finally, each surface residue was labeled as either a binding site residue, if it contacted another protein chain in the complex structure (< 4 Å non-H atom separation), or otherwise as a non-binding site residue.

Most machine learning classifier methods, include Random Forests, perform better on balanced input data that has a comparable number of positive and negative examples. Because of this, negative (non-binding site) examples were randomly chosen from the negative data such that there were an equal number of positive and negative examples in the training data. After training the Random Forest classifier on a balanced subset of the training data, predictions were made for all data in the (unbalanced) test set. The input data contained a total of 2391 positive examples for binding site residues.

### Random Forests

A Random Forest binary classifier was trained on the labeled residue data and used to predict whether or not each intramembrane surface residue is in a protein-protein binding site. The Random Forest method [29] was chosen because it is fast and achieves competitive accuracy on standard test classification problems. In addition, unlike the popular alternative methods of Support Vector Machines (SVMs) and Artificial Neural Networks (ANNs), it can utilize heterogeneous training data without rescaling and can also efficiently estimate the contribution of each variable to the prediction performance.

The overall prediction performance was evaluated by 10-fold cross-validation in which the data was randomly divided into 10 approximately equal size sets and predictions were made for each set in turn using a Random Forest trained on the data in the remaining 9 sets. The data was divided so that all residue data for a particular protein was contained entirely within one set. This insures that the predictions are made for a distinct set of proteins from those used to train the Random Forest classifier so that one obtains an accurate estimate of the prediction performance for novel data.

Briefly, a Random Forest is a set of decision trees in which the input data for each tree is randomized in two ways, by using a random subset of the total variables and by using a bootstrap sample of the data. The two main parameters in the method are the total number of trees and the number of variables per tree. Because the Random Forest generalization error converges to an asymptotic value as more trees are added, increasing the number of trees does not generally lead to worse overfitting [29]. For the binding site residue prediction, a total of 2000 trees in the Random Forest were found to be sufficient, since adding further trees did not significantly improve the prediction performance but increased the calculation time. Also the number of variables per tree was set at two because this gave the highest cross-validation accuracy. The accuracy showed little change upon varying this parameter. The prediction score, which varied from 0.0 to 1.0, was calculated as the fraction of decision trees classifying the data as a binding site.

The open source Random Forest implementation in the R [30] package randomForest [31] were used for all predictions. Statistical analysis was also performed in the R software environment.

### Prediction confidence

As in our previous studies [18,32], the confidence of the prediction for each residue was calculated from the score as the ratio of the class conditional likelihoods



The likelihoods in the numerator and denominator were calculated using Gaussian kernel density estimation of the scores in each respective class. A high value of R for a residue indicates that it is confidently predicted to be in a binding site, a low value indicates that it is confidently predicted to be outside of binding sites, and an intermediate value indicates an ambiguous prediction. The R values are useful for prioritizing the predictions before undertaking time-consuming and costly experimental validation.

## Results and Discussion

### Distinguishing characteristics of intramembrane protein-protein binding sites

Throughout this section we consider only the intramembrane portion of membrane protein complexes since the general properties of the solvated portions of the complexes, specifically both binding site and non-binding site surfaces, are expected to have similar properties to those of cytosolic proteins. The membrane core was defined to extend 15 Å in both directions perpendicular to the central membrane plane predicted by the TMDET method and available from the PDBTM database. The TMDET method accounts for both the protein backbone geometry and hydrophobicity in order to predict the extent and orientation of the membrane relative to the protein complex. TMDET uses the structure of the complex, and so incorporates the geometrical constraints that all transmembrane segments are delimited by two common membrane boundaries. This is an advantage over sequence-only prediction methods, when experimental structures are available.

Protein-protein binding sites on cytosolic proteins have different distributions of residue types on average than those on the exposed protein surface. Specifically protein-protein binding sites are enriched in large hydrophobic and uncharged polar residues and are depleted of charged residues [18,33]. This can be partially explained by the favorable solvation energy of burying hydrophobic residues and the unfavorable energy of burying charged residues in the interface.

A different trend in residue frequencies is expected for the intramembrane portion of membrane proteins because their surfaces are contacting the hydrocarbon tails of the lipid molecules comprising the membrane so that hydrophobic residues are energetically favorable on the exposed protein surface. Statistical tests using the benchmark set revealed that intramembrane protein-protein binding sites have higher frequencies of phenylalanine, tryptophan, and tyrosine residues and lower frequencies of valine residues than the remaining intramembrane protein surface (p < 0.05; Wilcoxon paired sign-rank tests with multiple testing corrections). In addition, residues occurring within protein-protein binding sites in membrane proteins have lower evolutionary rates, or equivalently higher conservation, than residues on the remaining intramembrane surface (*p *< 2.2 × 10^-16^, Wilcoxon rank sum test).

### Spatial Averaging of Scores

Our previous method for predicting protein-protein binding sites [18], as well as those of others [12-14,16], included the properties of neighboring residues in the training data. We found that this resulted in better performance than using only the properties of each individual residue. One explanation for the improved accuracy is that the binding sites are contiguous regions on the protein surface so that a given residue in a binding site is likely to be surrounded by other binding site residues. Likewise, surface residues outside of the binding sites are likely to be surrounded by other non-binding site residues. In other words, the binding site residues are spatially clustered and not randomly scattered about the surface. Including data for neighboring residues then provides additional independent information that improves the prediction accuracy.

We also investigated spatial averaging of Random Forest scores from predictions using only data from single residues and found that it gave slightly better performance than including properties for neighboring residues in training data (data not shown). For a given surface residue, the average score, *S*_*avg*_, was calculated as a linearly weighted average over the scores, *S*_*i*_, for all residues with C_α _separations from the central residue, *r*_*i*_, less than *r*_*max *_using



in which the summations are over all residues within the cutoff distance, *r*_*max*_. The score of the central residue has a weight of 1 while a residue at the cutoff distance would have the minimum weight, *w*_*min*_. Thus the scores for residues closest to the central one make a larger contribution to the average score *S*_*avg *_than those further away. The best values for the two adjustable parameters, which resulted in the highest AUC, were chosen by a grid search. The optimal values were found to be *r*_*max *_= 18 Å and *w*_*min *_= 0.1.

### Overall Prediction Accuracy

The overall prediction accuracy of the method was assessed by the area under the Receiver Operating Characteristic (ROC) curve for cross-validation results. The ROC curve is a plot of the sensitivity, or true positive rate, versus (1 - specificity), or false positive rate, and displays the tradeoff between these two quantities as the prediction score cutoff is varied. AUC can vary between 0.0 and 1.0. A value of 1.0 indicates perfect accuracy whereas a value near 0.5 indicates poor prediction performance.

**Figure 1:**
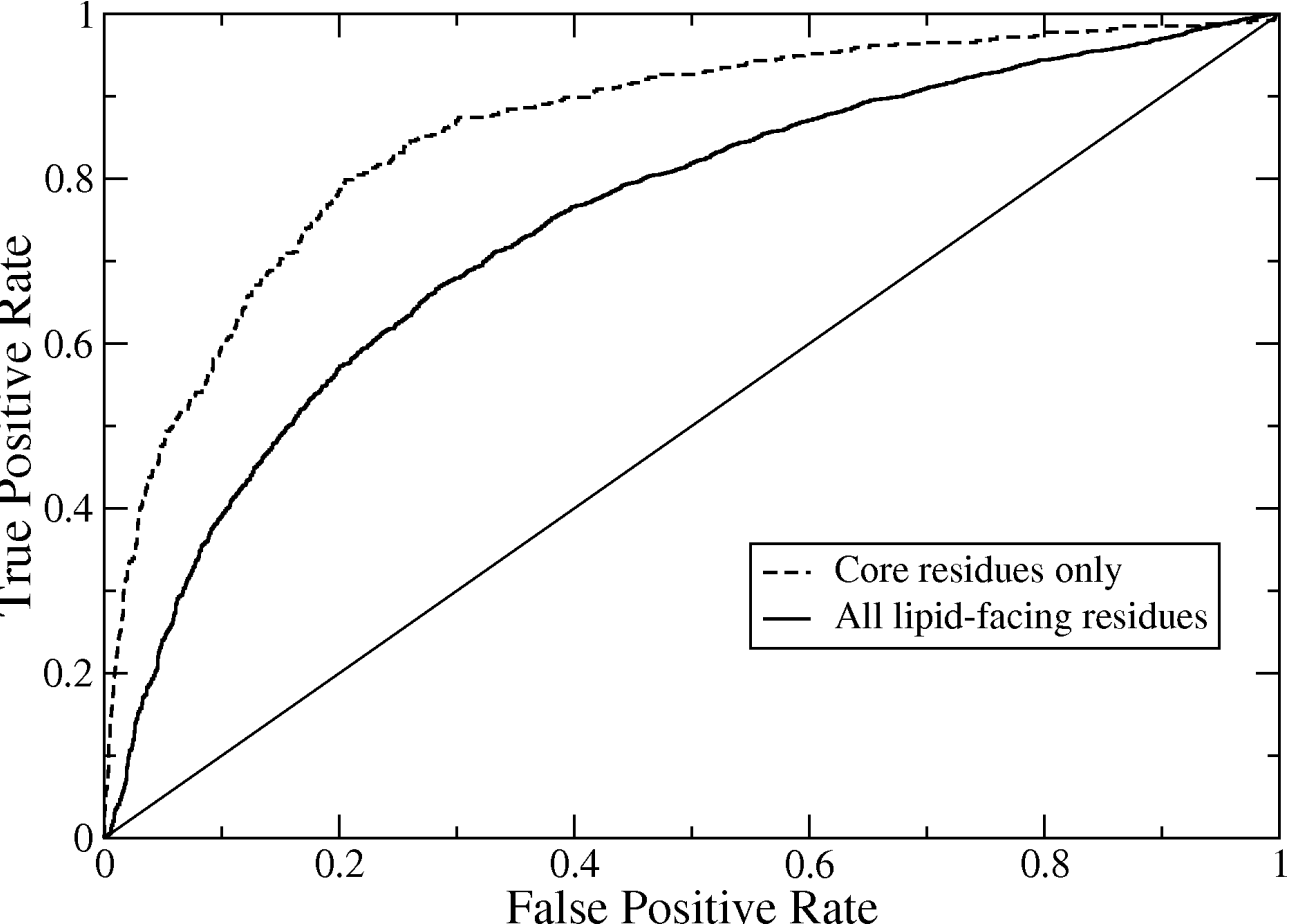
**Receiver operating characteristic (ROC) curves for the protein-protein binding site predictions**. The solid curve is for all intramembrane surface residues (AUC = 0.75) and the dashed curve is for only core residues, which are distant from the binding site boundary (AUC = 0.86).

The ROC curve for the cross-validation prediction results is shown in Figure 1. The AUC for this curve is 0.75. This is only slightly lower than the AUC value of 0.79 that we obtained for a set of non-membrane proteins using our previous method [18]. The similar prediction performance for membrane and non-membrane proteins shows that (1) there is a comparably strong signal in the training data that can be used to discriminate binding site and non-binding site residues and (2) there is sufficient training data for membrane proteins.

It is tempting to use the larger quantity of protein-protein binding site data for non-membrane proteins in order to train a predictor for membrane proteins. This was directly tested by training the same prediction method described above on data from a non-redundant set of 4296 non-membrane proteins, sharing less than 30% sequence identity, and making predictions for the membrane protein benchmark set data. The AUC for the prediction was only 0.36. This AUC value is actually less than the random expected value of 0.5 because the prediction results are anticorrelated, *i.e. *binding site residues are more often predicted as non-binding site residues and vice versa. One explanation of the anticorrelation is that whereas hydrophobic residues are more prevalent in non-membrane protein binding sites they are instead more prevalent on the lipid-exposed non-binding site surfaces of membrane proteins. Likewise, hydrophilic residues are more prevalent on the solvent-exposed non-binding site surface of non-membrane proteins whereas they are more prevalent in the protein-protein binding sites of membrane proteins. This result confirms the expectation that the different frequencies of surface residue types for membrane and non-membrane proteins, resulting from the different physiochemical environments of proteins in each class, implies that separate predictors trained on the same class of proteins (membrane or non-membrane) are required in order to achieve good prediction accuracy.

Also we found in our previous study [18] that central protein-protein binding site residues had higher prediction reliability than those near the periphery of the binding site. This was attributed to two factors: (1) the 14 nearest residues, whose properties are included in the training data, are more likely to also be within the binding site and so provide additional independent data to improve the prediction accuracy, (2) there is some ambiguity in the binding site boundary depending on how the binding site is defined (for example, based on loss of SASA upon forming a complex or intermolecular atomic contacts), and (3) central residues had greater evolutionary conservation than peripheral binding site residues, resulting in a stronger signal.

Here we define a core residue as one for which all other residues within a C_α_ separation distance of 8 Å belong to the same class (binding site residue or non-binding site residue). Thus core residues can be either inside or outside of the binding sites but are not near the binding site boundaries. The AUC for the core residues alone was 0.86, which is considerably higher than when residues near the binding site boundaries are included. This is consistent with the results of the earlier study, although that study only examined core residues within protein-protein binding sites. However, unlike that study, there was no significant difference in the evolutionary conservation between the core and peripheral (non-core) binding site residues. This implies that the last factor (#3), mentioned above, that contributes to improved prediction performance for core residues in cytosolic proteins does not contribute for membrane proteins. However, the remaining two factors (#1 and #2) probably also contribute to the improved accuracy for core residues in membrane proteins.

### Relative Importance of Residue Properties to Prediction Accuracy

**Figure 2:**
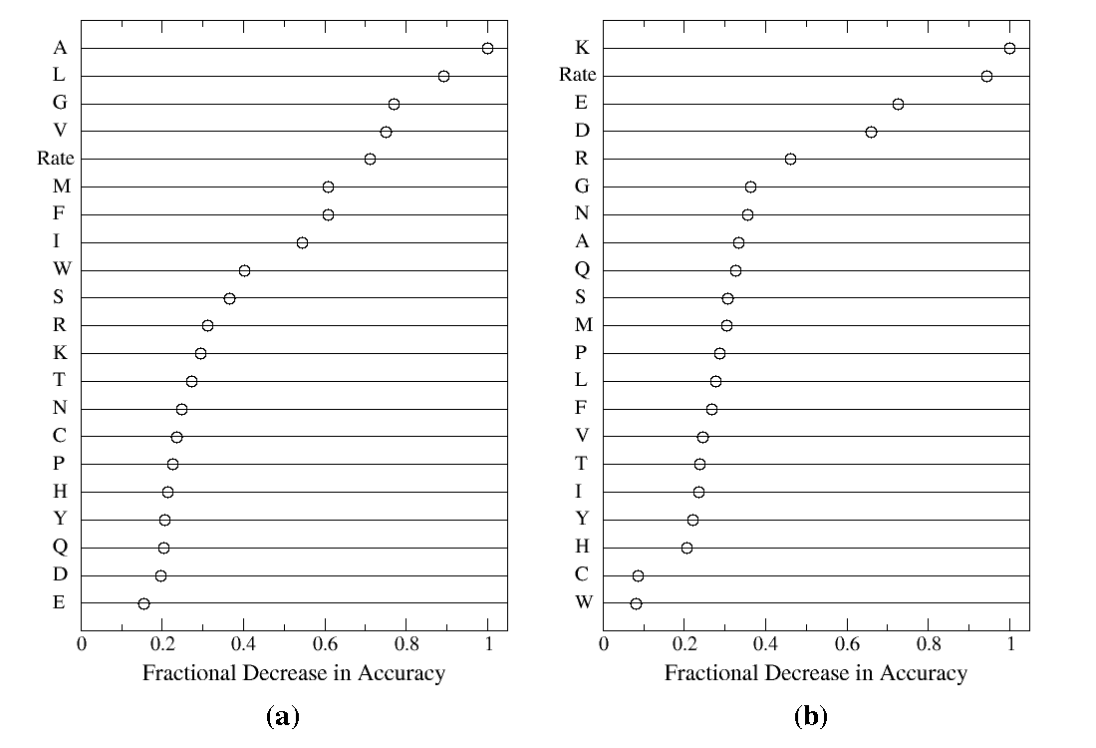
**The relative importance of each residue property to the overall accuracy for predicting protein-protein binding sites in (a) membrane proteins and (b) non-membrane proteins**. These properties are the frequencies of each residue type in a multiple sequence alignment of related proteins (indicated by the residue type abbreviation) and the evolutionary rate. A higher fractional decrease in accuracy indicates that the property contributes more to the prediction accuracy.

Because Random Forests use a bootstrap sample to train each classification tree, the remaining unused, or so-called out-of-bag, data can be used to obtain an estimate of each individual variable's contribution to the overall prediction accuracy [29]. This is accomplished by calculating the mean decrease in accuracy for the out-of-bag data upon randomly shuffling the values for the variable of interest. The results for membrane proteins are plotted in Figure 2a.

**Figure 3:**
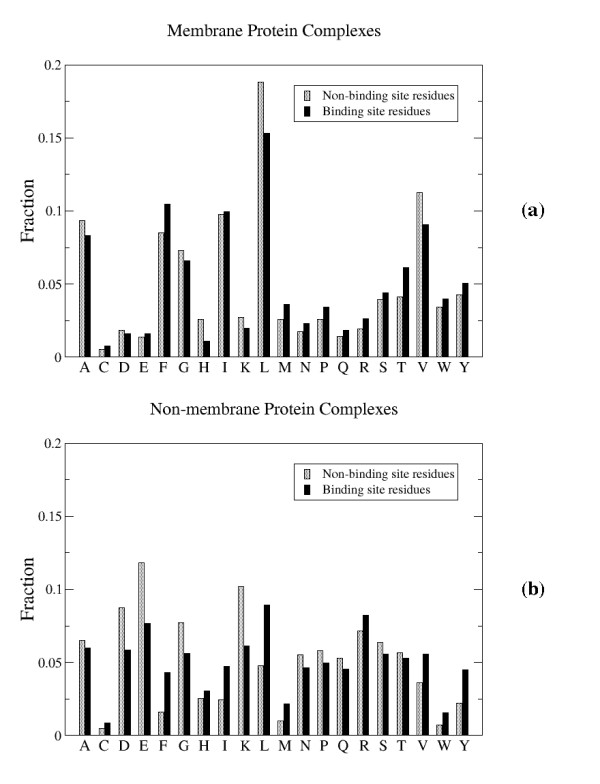
**Observed residue type frequencies in protein-protein binding sites and the remaining surface for (a) membrane and (b) non-membrane proteins**.

Comparison of Figure 2a with Figure 3a shows that the importance of each residue type is roughly correlated with its frequency of occurrence. The residue types contributing the most to the accuracy, namely alanine, leucine, glycine, and valine, are among the residues occurring most frequently in the intramembrane region of membrane proteins. Likewise, the least important residue types for the prediction, glutamic acid, aspartic acid, and glutamine, are some of the least frequently occurring residues in the membrane region. A similar comparison between figures 2b and 3b reveals the same trend for non-membrane proteins. Specifically, the residues contributing the most to accuracy, namely lysine, glutamic acid, aspartic acid, and arginine, are some of the most prevalent surface residues and the residues contributing the least to the prediction accuracy, tryptophan and cysteine, are the least frequently occurring surface residues in non-membrane proteins.

Although the quantity of training data for membrane proteins is considerably less than for non-membrane proteins, the fact that the importance of the column residue frequencies exhibit the same dependence on their frequencies of occurrence suggests that this trend is not due to a lack of sufficient data. Rather, the simplest interpretation of these trends is that the overall abundance of each residue type, which determines how prevalent the residue type is in the training data, generally dominates any differences in residue frequencies between each class (binding site and non-binding site residues). For example, even though the statistical tests showed that tyrosine residues are more prevalent in binding sites whereas leucine residues are not, the column frequencies of leucine residues are more important than those of tyrosine residues because the training data contains significantly more leucine residues, thus giving them a larger contribution to the overall prediction accuracy.

## Prediction Examples

We next briefly examine two examples in which the protein-protein binding site predictions aid in identifying or confirming the correct biologically relevant complex from X-ray structures. Again, cross-validation predictions, in which the predictor was trained on data for dissimilar protein complexes, were used in order to provide a realistic assessment of the prediction performance.

**Figure 4:**
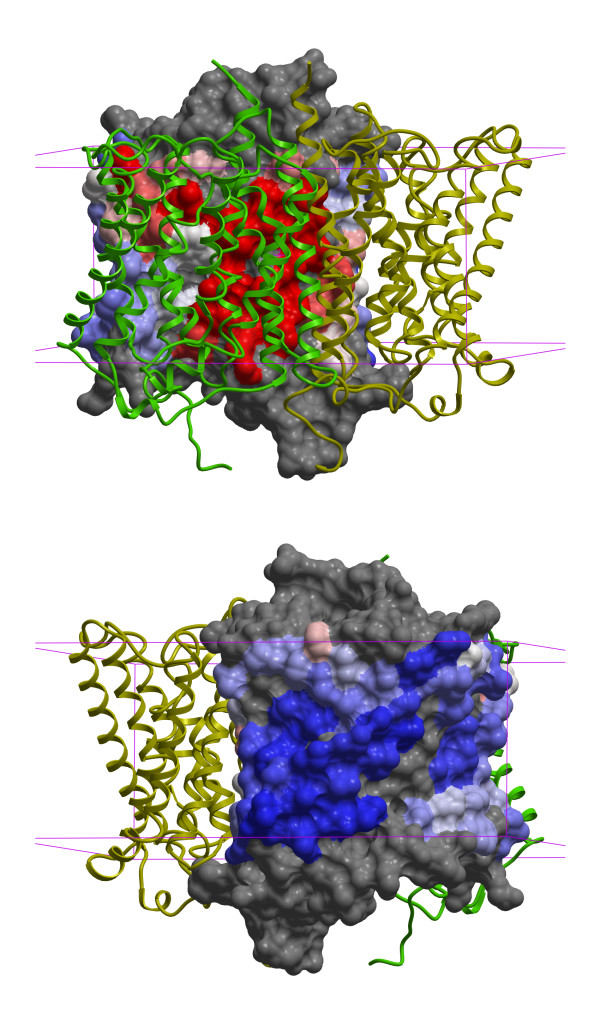
**Successful prediction of protein-protein binding site residues for a bacterial homolog of human Rhesus protein Rh50 (PDB entry **3B9W**)**. The protein forms a homotrimeric complex; two subunits are shown in green and yellow ribbon representation. The surface of one subunit is colored according to the probability ratio for each surface residue. The colors vary continuously from blue to white to red for low, neutral (1.0), and high ratios, respectively. The two figures differ by a 180° rotation about the vertical axis. The red high confidence predicted binding site residues are correctly located in the actual binding site and the blue high confidence non-binding site residues are located on the opposite face. The boundaries of the membrane are shown in purple.

Figure 4 shows the protein-protein binding site prediction for an ammonium transport (Amt) protein from *Nitrosomonas europaea *(PDB entry 3B9W[34]). This NeRH50 protein is a rare bacterial homolog of the human Rhesus (Rh) group antigens, RhD and RhCE, and Rh-associated glycoprotein RhAG. Along with the ABO system, the Rh antigens are the most clinically important antigens in blood transfusions. The authors who determined the Amt structure concluded that it is homotrimeric, based on their observation of a tightly packed trimer generated by the threefold crystallographic symmetry axis. Because of the homology between Amt and human Rh50 proteins they suggested that the Rh50 are also homotrimeric, in contrast with earlier experimental studies that concluded that they likely form heterotetramers with RhD or RhCE [35,36]. As can be seen in the figure, the confidently predicted binding residues on one subunit agree with the interfaces in the proposed trimeric biological complex, thus confirming the conclusion of Ref. [34] for the NeRH50 complex. Importantly, the remaining protein surface outside the binding site is also confidently predicted not to contain other binding site residues. Binding site predictions were also made for homology models of RhD, RhCE, and RhD obtained from MODBASE. They showed qualitatively similar results to those for NeRH50, except for a slightly larger predicted binding patch (data not shown). The similar results are probably due to the relatively high sequence similarity (~30-35%) between the human proteins and NeRH50. Although the binding site predictions do not rule out a tetrameric human Rh complex they provide a prioritized list of potential binding site residues that can be experimentally tested.

**Figure 5:**
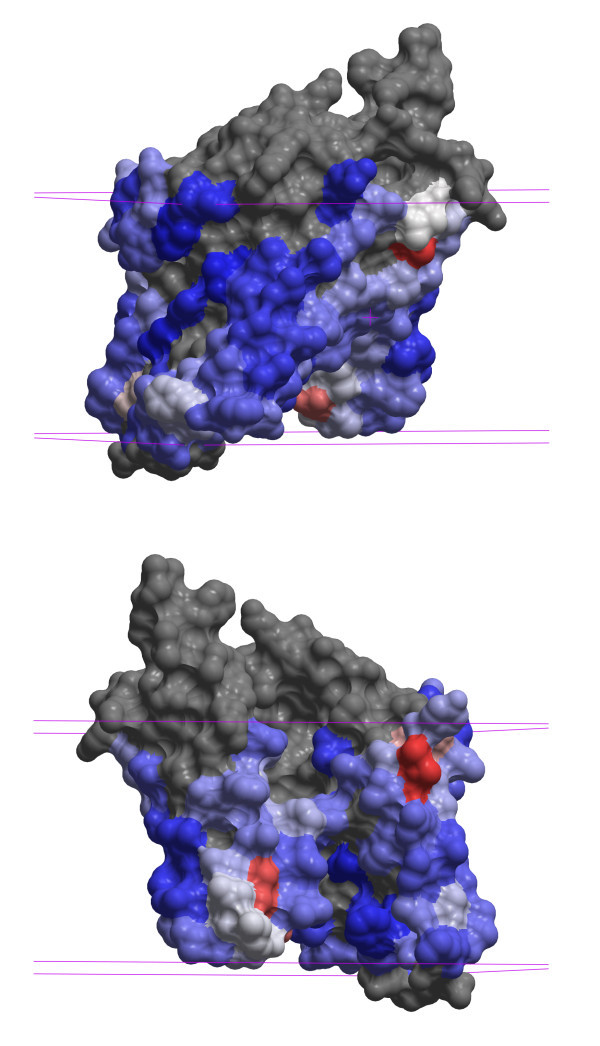
**Protein-protein binding predictions for an archaeal Site-2 Protease (S2P) family intramembrane metalloprotease (PDB entry **3B4R**)**. The representation and coloring scheme are the same as in Figure 4. Because no binding site of sufficient size is predicted in the intramembrane region the dimer in the X-ray structure is likely to be an artifact of the crystal environment and the protease is predicted to function as a monomer.

Figure 5 shows the binding site prediction for an archaeal Site-2 Protease (S2P) family intramembrane metalloprotease (PDB entry 3B4R[37]). Although a few isolated residues near the membrane-solvent interface have high scores, there are no contiguous predicted binding patches on the intramembrane surface. This implies that the protein is likely to be a monomer and not a homodimer, as the BIOMT annotation indicates. The substrate peptide binding site does not appear in the prediction because it is buried in this presumably closed conformation of the protease. This example also suggests that post-processing to remove small predicted binding site patches, as was done for non-membrane proteins in Ref. [18], would remove spurious binding patches, *e.g. *small molecule binding sites, that are too small to be protein binding sites, and so may improve prediction accuracy.

## Conclusion

The protein-protein binding site prediction method for membrane proteins described in this study was found to yield accuracy that was comparable to that for non-membrane proteins. Although there are considerably fewer experimental structures of membrane proteins than non-membrane proteins, because the predictions are made for individual surface residues there is a sufficient quantity of independent examples for training a Random Forest classifier that gives accurate results. Also, as expected from the different occurrence frequencies of surface residue types in membrane and non-membrane proteins, a predictor trained on non-membrane proteins gave poor accuracy when applied to membrane proteins. Thus separate predictors for membrane and non-membrane proteins are needed. In addition, a prediction procedure that is different than the ones used in previous studies was found to give better accuracy. Random Forest predictions were first made for individual surface residues and then the resulting scores of nearby residues were averaged in order to arrive at the final prediction score. Predictions could not be made for some proteins due to an insufficient number of related protein sequences needed for the multiple sequence alignment, however this is expected to improve with the rapidly growing number of available protein sequences.

The prediction method presented here is expected to have applications in guiding experimental investigations of membrane protein interactions and also in the prediction of protein complex structures using computational methods such as docking or threading. In addition to these applications, several future areas of investigation are possible. First, because the method relies only on residue-level information, it is expected to give accurate results for homology models, which are generally correct for regions with well-defined secondary structure but often have errors in loops or side chain conformations. A study of the prediction accuracy for homology models of varying quality would help quantify what accuracy can be expected. Second, because the method relies on a multiple sequence alignment of similar sequences, the choice of included sequences can affect the final prediction accuracy. The implicit assumption that the proteins with sequences in the multiple alignment have the same protein-protein binding site, may be incorrect, particularly if distantly related sequences are included. It would be useful to have a method for selecting the optimal set of sequences to include in the alignment. Finally, contiguous binding patches could be calculated from the individual residue predictions. This would then give a lower bound on the number of independent binding sites on the protein surface.

## Authors' contributions

AJB designed and performed the study, analyzed the results, and wrote the manuscript.

## Supplementary Material

Additional file 1 **Benchmark set of membrane protein complexes**. This zip archive contains two files. One is a tab-separated table file with information on the PDB structures of membrane protein complexes used in this study. The other is a PDF file that provides a detailed description of the table format. Click here for file

## References

[B1] Bakheet TM, Doig AJ (2009). Properties and identification of human protein drug targets. Bioinformatics.

[B2] Yildirim MA, Goh KI, Cusick ME, Barabasi AL, Vidal M (2007). Drug-target network. Nat Biotechnol.

[B3] Elofsson A, von Heijne G (2007). Membrane protein structure: prediction versus reality. Annu Rev Biochem.

[B4] Bordner AJ, Gorin AA (2008). Comprehensive inventory of protein complexes in the Protein Data Bank from consistent classification of interfaces. BMC Bioinformatics.

[B5] Ofran Y, Rost B (2003). Predicted protein-protein interaction sites from local sequence information. FEBS Lett.

[B6] Yan C, Dobbs D, Honavar V (2004). A two-stage classifier for identification of protein-protein interface residues. Bioinformatics.

[B7] Res I, Mihalek I, Lichtarge O (2005). An evolution based classifier for prediction of protein interfaces without using protein structures. Bioinformatics.

[B8] Chen XW, Jeong JC (2009). Sequence-based prediction of protein interaction sites with an integrative method. Bioinformatics.

[B9] Jones S, Thornton JM (1997). Prediction of protein-protein interaction sites using patch analysis. J Mol Biol.

[B10] Landgraf R, Xenarios I, Eisenberg D (2001). Three-dimensional cluster analysis identifies interfaces and functional residue clusters in proteins. J Mol Biol.

[B11] Neuvirth H, Raz R, Schreiber G (2004). ProMate: a structure based prediction program to identify the location of protein-protein binding sites. J Mol Biol.

[B12] Zhou HX, Shan Y (2001). Prediction of protein interaction sites from sequence profile and residue neighbor list. Proteins.

[B13] Fariselli P, Pazos F, Valencia A, Casadio R (2002). Prediction of protein--protein interaction sites in heterocomplexes with neural networks. Eur J Biochem.

[B14] Wang B, Chen P, Huang DS, Li JJ, Lok TM, Lyu MR (2006). Predicting protein interaction sites from residue spatial sequence profile and evolution rate. FEBS Lett.

[B15] Chen H, Zhou HX (2005). Prediction of interface residues in protein-protein complexes by a consensus neural network method: test against NMR data. Proteins.

[B16] Koike A, Takagi T (2004). Prediction of protein-protein interaction sites using support vector machines. Protein Eng Des Sel.

[B17] Bradford JR, Westhead DR (2005). Improved prediction of protein-protein binding sites using a support vector machines approach. Bioinformatics.

[B18] Bordner AJ, Abagyan R (2005). Statistical analysis and prediction of protein-protein interfaces. Proteins.

[B19] Burgoyne NJ, Jackson RM (2006). Predicting protein interaction sites: binding hot-spots in protein-protein and protein-ligand interfaces. Bioinformatics.

[B20] Sikic M, Tomic S, Vlahovicek K (2009). Prediction of protein-protein interaction sites in sequences and 3D structures by Random Forests. PLOS Comp Biol.

[B21] Tusnady GE, Dosztanyi Z, Simon I (2004). Transmembrane proteins in the Protein Data Bank: identification and classification. Bioinformatics.

[B22] Tusnady GE, Dosztanyi Z, Simon I (2005). PDB_TM: selection and membrane localization of transmembrane proteins in the protein data bank. Nucleic Acids Res.

[B23] Krissinel E, Henrick K (2007). Inference of macromolecular assemblies from crystalline state. J Mol Biol.

[B24] White SH, Wimley WC (1998). Hydrophobic interactions of peptides with membrane interfaces. Biochim Biophys Acta.

[B25] Altschul SF, Madden TL, Schaffer AA, Zhang J, Zhang Z, Miller W, Lipman DJ (1997). Gapped BLAST and PSI-BLAST: a new generation of protein database search programs. Nucleic Acids Res.

[B26] Li W, Godzik A (2006). Cd-hit: a fast program for clustering and comparing large sets of protein or nucleotide sequences. Bioinformatics.

[B27] Edgar RC (2004). MUSCLE: multiple sequence alignment with high accuracy and high throughput. Nucleic Acids Res.

[B28] Bordner AJ, Abagyan R (2005). REVCOM: a robust Bayesian method for evolutionary rate estimation. Bioinformatics.

[B29] Breiman L (2001). Random forests. Machine Learning.

[B30] R Development Core Team (2009). R: A language and environment for statistical computing. Vienna, Austria.

[B31] Liaw A, Wiener M (2002). Classification and regression by randomForest. R News.

[B32] Bordner AJ (2008). Predicting small ligand binding sites in proteins using backbone structure. Bioinformatics.

[B33] Jones S, Thornton JM (1996). Principles of protein-protein interactions. Proc Natl Acad Sci USA.

[B34] Lupo D, Li XD, Durand A, Tomizaki T, Cherif-Zahar B, Matassi G, Merrick M, Winkler FK (2007). The 1.3-A resolution structure of Nitrosomonas europaea Rh50 and mechanistic implications for NH3 transport by Rhesus family proteins. Proc Natl Acad Sci USA.

[B35] Eyers SA, Ridgwell K, Mawby WJ, Tanner MJ (1994). Topology and organization of human Rh (rhesus) blood group-related polypeptides. J Biol Chem.

[B36] Hartel-Schenk S, Agre P (1992). Mammalian red cell membrane Rh polypeptides are selectively palmitoylated subunits of a macromolecular complex. J Biol Chem.

[B37] Feng L, Yan H, Wu Z, Yan N, Wang Z, Jeffrey PD, Shi Y (2007). Structure of a site-2 protease family intramembrane metalloprotease. Science.

