# Supplementary material for: Predicting protein-protein binding sites in membrane proteins
Source: BMC Bioinformatics. 2009 Sep 24;10:312. doi: 10.1186/1471-2105-10-312 (PMC2761413; doi:10.1186/1471-2105-10-312)
Supplement: Additional file 1 — Benchmark set of membrane protein complexes. This zip archive contains two files. One is a tab-separated table file with information on the PDB structures of membrane protein complexes used in this study. The other is a PDF file that provides a detailed description of the table format. [file 1471-2105-10-312-S1.zip › Table Description.pdf]

Description of the membrane protein benchmark set table

The tab-separated table file contains information on all structures included in the non-redundant set of membrane protein complexes. The table columns are:

- "A-alpha or B-beta": A denotes an α-helical membrane protein complex and B denotes a β-barrel complex.
- "PDB entry": Protein Data Bank (PDB) entry ID
- "Resolution": Resolution of the PDB X-ray structure in Å.
- "Biological complex": The chain names and crystallographic symmetry transformations necessary to generate the biological protein complex. A comma-separated list of chain names precedes the colon and an ampersand-separated list of transformations follows the colon. Except for the trivial identity transformation, denoted by "1", the transformations are denoted as x',y',z', in which the primed coordinates are the new (transformed) coordinates expressed in terms of the original coordinates.
- "Identical chains": A semicolon-separated list of groups of chain names for identical proteins. Within each group the identical chain names are separated by commas.
- "Predicted chains": A list of all chains for which protein-protein binding site predictions were made. All of these chains satisfied the requirement that a sufficient number of similar amino acid sequences (≥ 20) could be collected.
